# Genetic analysis of production, physiological, and egg quality traits in heat-challenged commercial white egg-laying hens using 600k SNP array data

**DOI:** 10.1186/s12711-019-0474-6

**Published:** 2019-06-25

**Authors:** Kaylee Rowland, Chris M. Ashwell, Michael E. Persia, Max F. Rothschild, Carl Schmidt, Susan J. Lamont

**Affiliations:** 1 Department of Animal Science, Iowa State University, Ames, USA; 2 Prestage Department of Poultry Science, North Carolina State University, Raleigh, USA; 3 Department of Animal and Poultry Sciences, Virginia Tech, Blacksburg, USA; 4 University of Delaware, Animal and Food Sciences, Newark, USA

## Abstract

**Background:**

Heat stress negatively affects the welfare and production of chickens. High ambient temperature is considered one of the most ubiquitous abiotic environmental challenges to laying hens around the world. In this study, we recorded several production traits, feed intake, body weight, digestibility, and egg quality of 400 commercial white egg-laying hens before and during a 4-week heat treatment. For the phenotypes that had estimated heritabilities (using 600k SNP chip data) higher than 0, SNP associations were tested using the same 600k genotype data.

**Results:**

Seventeen phenotypes had heritability estimates higher than 0, including measurements at various time points for feed intake, feed efficiency, body weight, albumen weight, egg quality expressed in Haugh units, egg mass, and also for change in egg mass from prior to heat exposure to various time points during the 4-week heat treatment. Quantitative trait loci (QTL) were identified for 10 of these 17 phenotypes. Some of the phenotypes shared QTL including Haugh units before heat exposure and after 4 weeks of heat treatment.

**Conclusions:**

Estimated heritabilities differed from 0 for 17 traits, which indicates that they are under genetic control and that there is potential for improving these traits through selective breeding. The association of different QTL with the same phenotypes before heat exposure and during heat treatment indicates that genomic control of traits under heat stress is distinct from that under thermoneutral conditions. This study contributes to the knowledge on the genomic control of response to heat stress in laying hens.

**Electronic supplementary material:**

The online version of this article (10.1186/s12711-019-0474-6) contains supplementary material, which is available to authorized users.

## Background

Heat stress negatively affects the welfare and production of chickens worldwide. Heat is considered one of the most ubiquitous environmental challenges to laying hens around the world [1]. Numerous studies have reported a consistent decrease in feed intake, body weight, egg production, egg quality, and feed efficiency after exposure to high environmental temperatures [2–5]. Lower egg production and lower egg quality mean that food security and food safety for the global human population are also negatively impacted by heat stress in laying hens [6].

Many of the studies that have reported the effects of high ambient temperature on the losses in production in broilers and layers have been reviewed by [6]. However, few studies have investigated the genetic component of the response to high ambient temperature in laying hens. Mack et al. [7] demonstrated genetic differences in production and behavior traits during a heat challenge between two genetically and phenotypically distinct lines of White Leghorns (DeKalb XL and KGB), which opens the door for identifying genomic regions or variants that impact layer production under high ambient temperature.

In this study, we exposed white egg-laying hens to a 4-week heat challenge. Egg production, feed intake, body weight, digestibility, and egg quality traits were recorded before exposure to heat and at multiple time points during exposure to heat to quantify changes in these phenotypes. Heritabilities were estimated and associations between these traits and single nucleotide polymorphisms (SNPs) were tested using genotype information from the Axiom Chicken 600k Genotyping SNP Array [11] (Thermo Fisher Scientific, Inc., Waltham, MA, USA). Understanding the genetic control of response to heat exposure should contribute to the implementation of selective breeding to produce chickens that are more tolerant to heat stress.

## Methods

### Animals, husbandry, and heat treatment

Hy-Line W-36 female parent line chicks were reared at Hy-Line International (Dallas Center, IA) until 18 weeks of age. For this study, we used 400 pullets that were transported from Hy-Line International to Virginia Tech (Blacksburg, VA). The birds were transferred into pullet transportation coops, loaded into a long haul livestock trailer that was outfitted with ventilation fans, transported overnight, and immediately transferred to individual cages in an environmentally-controlled room in order to reduce as much as possible exposure to high temperatures during transportation. Four cages (length 38.1 cm × width 22.9 cm $\times$ height 43.2 cm) were stacked on one of three levels in each bank with the resulting 12 cages placed on wheels so that they could be transported from pre-heat to heat treatment chambers. Temperature was maintained at 23 °C until 24 weeks of age for acclimation. Birds were allowed ad libitum access to a mash layer diet and water. The diet contained 0.20% titanium dioxide as a marker for the calculation of apparent metabolizable energy (AMEn).

At the beginning of the heat treatment, each battery cage bank was split into two, and each of these was placed into pre-heated rooms (N = 200 hens, each) that received the same treatment. Heat treatment began at 24 weeks of age and continued until 28 weeks of age, i.e. during 4 weeks. The profile of the daily heat cycle, beginning at 9:00 am, was 7 h at 35 °C and then at 30 °C for the remaining 17 h.

### Phenotypes

Eggs were collected each day between 9:00 and 10:00 am. Egg weight, egg production, and egg mass were recorded individually each day and averaged over 2-week periods: the 2 weeks before initiating the heat treatment, the first 2 weeks of heat treatment, and the last 2 weeks of heat treatment. Feed intake was recorded for the same 2-week periods by weighing the feed added each day and weighing the feed remaining at the end of each 2-week period. Egg quality measurements (Haugh units, albumen weight, yolk weight, shell weight, and shell thickness) were recorded 1 day before exposure to heat, then 2 days, 1, 2, 3, and 4 weeks after initiating the heat treatment. Haugh units were measured with a Mattox and Moore Haugh meter. After determining the Haugh unit of each egg, the yolk was separated from the albumen and their weights were recorded. To quantify shell thickness, shells were left to dry overnight at room temperature, then three individual measurements of each egg’s thickness were recorded with a micrometer and an average value was calculated per egg. Cloacal body temperature measurements and sampling of fecal material for calculation of AMEn, were performed 1 day before exposure to heat, three to five hours after initiating the heat treatment on the first day, and 2 and 4 weeks after initiating the heat treatment. Gross energy (kcal/g), nitrogen (g), and titanium (%) contents were quantified from feed and fecal samples to calculate AMEn [8] using the following equations [9]:

$$\begin{aligned} & AMEn\,per\,g\, diet = gross\, energy\, of\, feed - (fecal\, energy\, per\, g \,diet + 8.22 \times nitrogen \,retained\, per\, g\, diet, \\ & Fecal \,energy \,per\, g\, diet = gross\, energy\, of\, fecal \times \left( {\frac{{\text{TiO}_{2} \,in\, diet }}{{\text{TiO}_{2} \, in \,fecal}}} \right), \\ & Nitrogen\, retained\, per\, g \,diet = nitrogen \,per\, g \,diet\, - nitrogen\, per\, g \,fecal\, \times \left( {\frac{{\text{TiO}_{2} \, in\, diet}}{{\text{TiO}_{2} \, in \,fecal}}} \right). \\ \end{aligned}$$

The terms acute and chronic refer, respectively, to the first measurement of a trait after the initiation of heat treatment and the later measurements of the same trait. The specific time of measurement of the acute traits varies with the phenotype.

Blood was collected before exposure to heat and after initiating the heat treatment for in-depth analysis of blood gas and chemistry components. The genetic analysis of these traits is described in a companion manuscript [10].

### Genotyping and quality control

Whole blood was collected from the 400 pullets at 17 weeks of age and shipped to GeneSeek, Neogen Genomics (Lincoln, NE, United States). Genomic DNA was isolated and used for genotyping with the Axiom Chicken 600k Genotyping SNP Array [11] (Thermo Fisher Scientific, Inc., Waltham, MA, USA). Axiom Chicken Genotyping Array annotation files, release 35, were based on the galGal genome version 5.0 (Thermo Fisher Scientific). The following quality filtering criteria, which are described in the Axiom Analysis Suite User Guide obtained from Thermo Fisher Scientific [12], were applied: call rate (CR) ≥ 95, minor allele frequency (MAF) ≥ 0.01, Fisher’s linear discriminant (FLD) ≥ 4, homozygote ratio offset (HomRO) ≥ − 0.6, BB cluster variance X (BB.varX) ≤ 0.9, BB cluster variance Y (BB.varY) ≤ 0.45, AB cluster variance X (AB.varX) ≤ 0.55, AB cluster variance Y (AB.varY) ≤ 0.5, AA cluster variance X (AA.varX) ≤ 0.6, homozygous Fisher’s linear discriminant (HomFLD) ≥ 9, heterozygous strength offset (HetSO) ≥ − 0.2, and conversion type ≠ off-target variant (“OTV”). After these filtering steps, 261,509 SNPs and 374 animals remained for analyses.

### Data analyses

For all traits, differences between all the measurements performed before exposure to heat and after initiating the heat treatment were calculated to determine the change in each phenotype caused by heat across time. These values were used for estimating heritabilities and the association analysis.

Heritabilities and variance components were estimated using ASReml 4.0 [13] with a univariate animal model:

$$Y_{ij} = \mu + FE_{i} + A_{j} + e_{ij} ,$$

$Y_{ij}$ is the dependent variable of each phenotype (phenotypes are listed in Tables 1, 2 and 3), $j$ is animal and $i$ is cage row within a room.

**Table 1:** Heritability (standard error) estimates for egg quality and body weight traits

Trait	Pre-heat	Acute^a^	Week 1	Week 2	Week 3	Week 4
Albumen weight	0.15 (0.10)	0.39 (0.15)	0.42 (0.15)	0.23 (0.11)	0.19 (0.13)	0.05 (0.11)
Haugh units	0.26 (0.11)	0.15 (0.13)	0.59 (0.14)	0.24 (0.11)	0.07 (0.1)	0.40 (0.13)
Shell thickness	NC^b^	0.28 (0.15)	0.14 (0.14)	0.05 (0.1)	NC^b^	0.22 (0.16)
Shell weight	0.02 (0.1)	0.03 (0.12)	0.11 (0.15)	0.20 (0.11)	0.21 (0.13)	0.29 (0.15)
Yolk weight	0.09 (0.10)	0.08 (0.15)	0.16 (0.14)	0.06 (0.1)	0.09 (0.11)	0.11 (0.11)
Body weight	0.35 (0.11)	NA^c^	NA^c^	0.44 (0.10)	0.31 (0.10)	0.37 (0.11)

^a^Eggs collected the morning after the first heat cycle, such that they were formed during the first heat cycle

^b^Does not converge

^c^Trait not measured at this time point

**Table 2:** Heritability (standard error) estimates for physiological traits

Trait	Pre-heat	Acute^a^	Week 2	Week 4
AMEn^b^	0.10 (0.10)	0.17 (0.10)	0.19 (0.13)	0.24 (0.13)
Body temperature	NC^c^	0.05 (0.09)	NC^c^	0.13 (0.1)

^a^First day of heat exposure

^b^Apparent metabolizable energy

^c^Does not converge

**Table 3:** Heritability (standard error) estimates for production traits

Trait	2 weeks pre-heat^a^	Weeks 1–2	Weeks 3–4
Egg production	0.06 (0.1)	0.03 (0.09)	NC^b^
Egg mass	0.43 (0.11)	0.30 (0.10)	0.24 (0.10)
Egg weight	0.05 (0.09)	0.16 (0.1)	0.23 (0.12)
Feed intake	0.18 (0.11)	0.31 (0.11)	0.17 (0.1)
Feed efficiency (g feed/g egg)	NC^b^	0.23 (0.11)	0.13 (0.1)

^a^Phenotypes are an average over 2-week periods

^b^Does not converge

A fixed effect for cage row within the room ($FE_{i}$) was included if the effect on the phenotype was significant, which was the case for body temperature only. Animal genetic effects ($A_{j}$) with a genomic relationship matrix computed from SNP genotypes, as described by [14], and residual effects ($e_{ij}$) were the two random effects. Heritabilities were defined as different from 0 when they were more than two times the standard error.

Only the traits that had an estimated heritability different from 0 were used in the association analyses, since a heritability not different from 0 indicates absence of a genetic component and thus association analysis is not appropriate. Association analyses were performed using a hierarchical generalized linear model (same effects as described for the estimation of heritabilities) [15] in GenABEL [16]. The association analysis method used in GenABEL, polygenic hglm and mmscore, is similar to the FASTA method used for related individuals as described by [17].

To determine the number of independent tests, we used a modified Bonferroni multiple test correction, previously described in [18], and found 16,085 independent tests. The 20% genome-wide threshold was calculated to be 1.2 $\times$ 10^−5^.

## Results

### Heritability

Seventeen phenotypes had heritability estimates higher than 0: feed intake (2 weeks after initiating the heat treatment (hereafter termed as post-heat), 0.31), feed efficiency (2 weeks post-heat, 0.23), body weight (before exposure to heat (hereafter termed pre-heat), 0.35; 2 weeks post-heat, 0.44; 3 weeks post-heat, 0.31; 4 weeks post-heat, 0.37), albumen weight (acute heat, 0.39; 1 week post-heat, 0.42; 2 weeks post-heat, 0.23), Haugh units (pre-heat, 0.26; 1 week post-heat, 0.59; 2 weeks post-heat, 0.24; 4 weeks post-heat, 0.40), egg mass (pre-heat, 0.43; 2 weeks post-heat, 0.30; 4 weeks post-heat, 0.24), and change in egg mass from prior to heat exposure to 4 weeks after initiation of heat exposure (Tables 1, 2 and 3). This last trait was the only one calculated as a change before and after heat treatment that had a measureable heritability, 0.19 ± 0.09 (data not shown for the other traits).

### Associations between quantitative trait loci and phenotypes

**Fig. 1:**
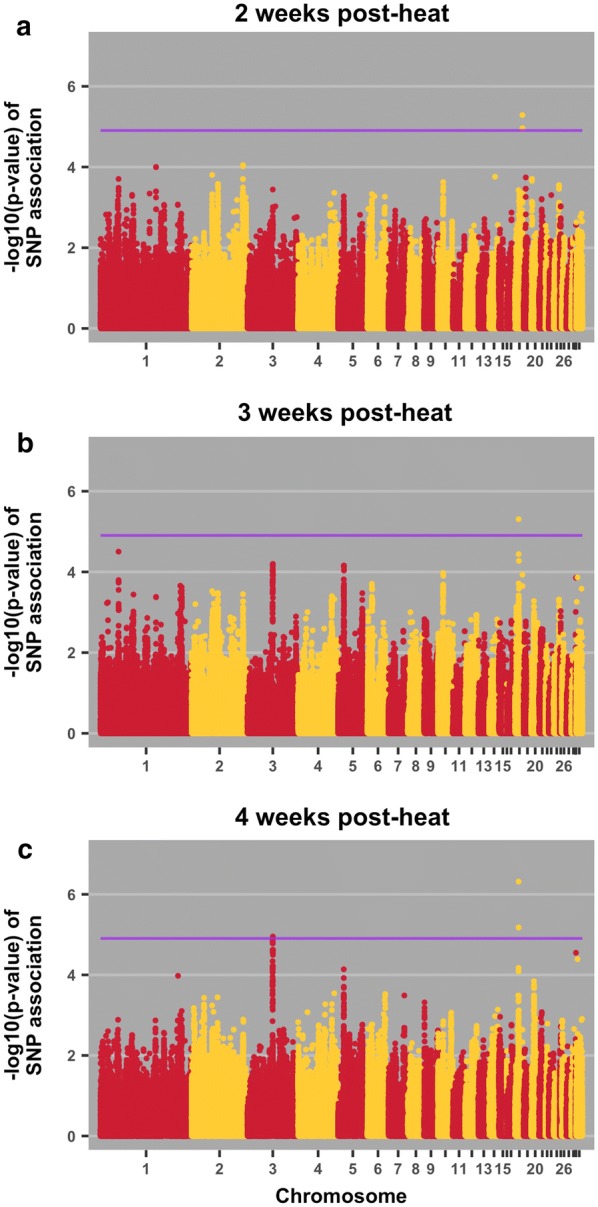
Manhattan plots for body weight 2 weeks (**a**), 3 weeks (**b**), and 4 weeks (**c**) post-heat initiation. The purple line indicates the 20% genome-wide threshold

**Fig. 2:**
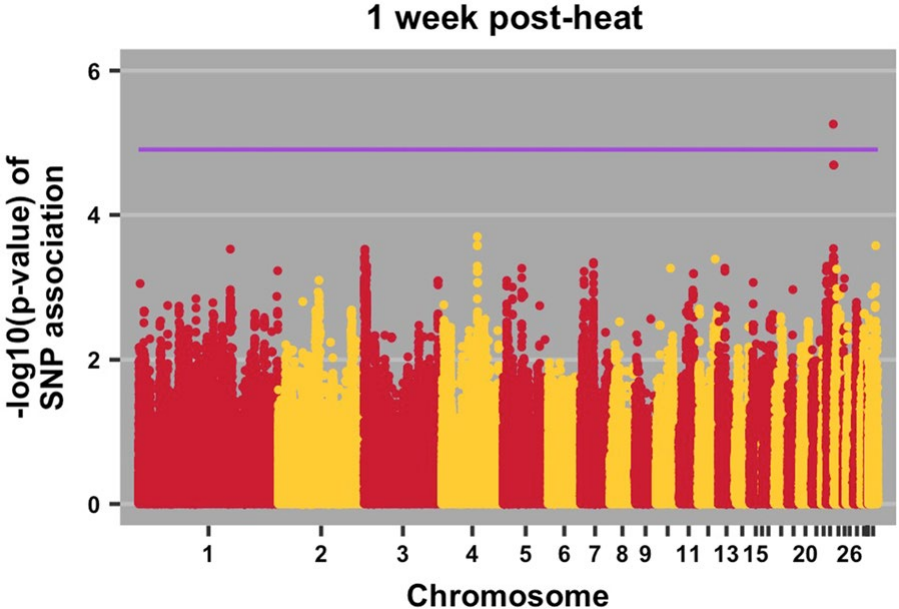
Manhattan plot for albumen weight 1 week post-heat initiation. The purple line indicates the 20% genome-wide threshold

**Fig. 3:**
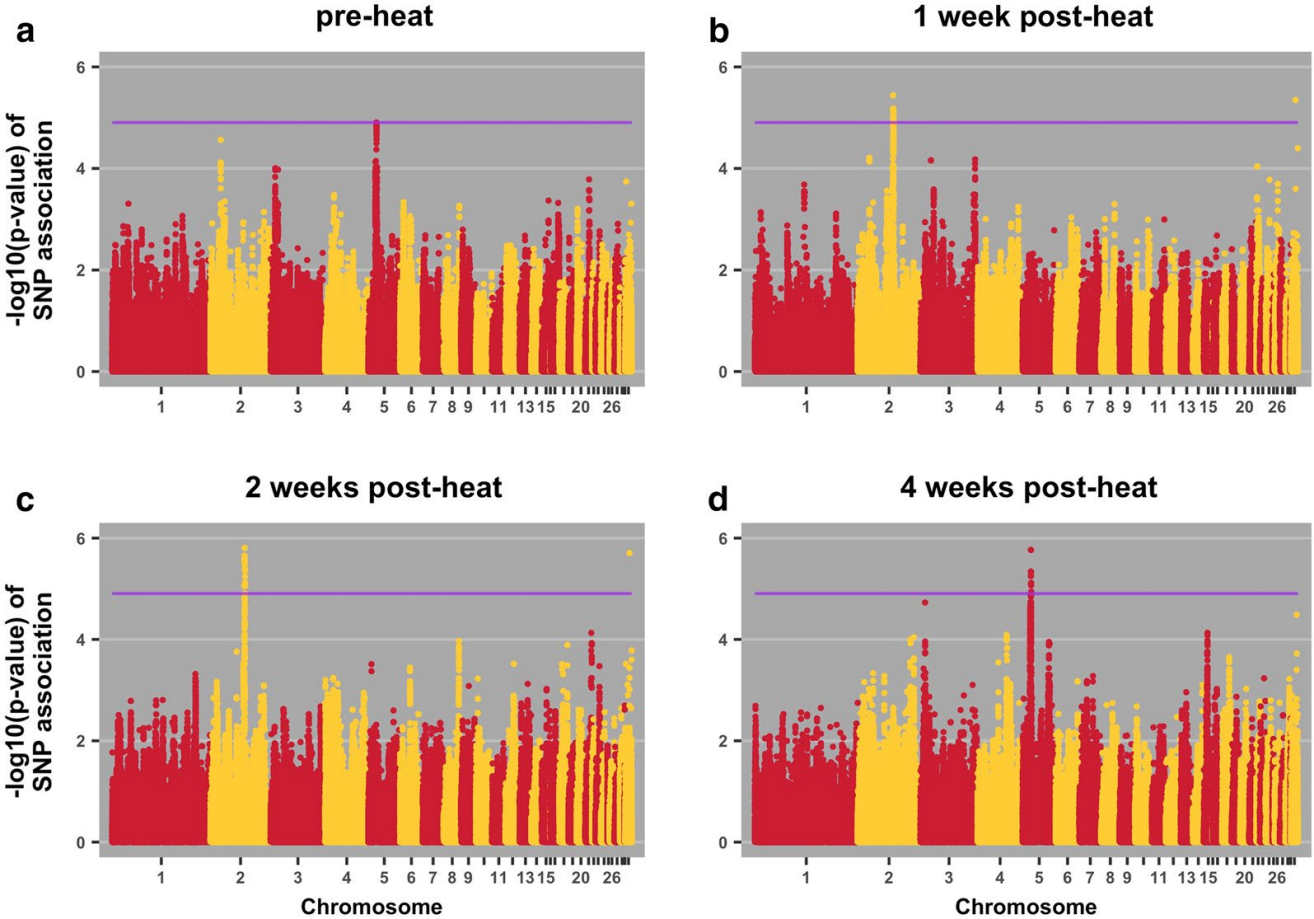
Manhattan plots for Haugh units pre-heat (**a**), 1 week post-heat (**b**), 2 weeks post-heat (**c**), and 4 weeks post-heat (**d**). The purple line indicates the 20% genome-wide threshold

**Fig. 4:**
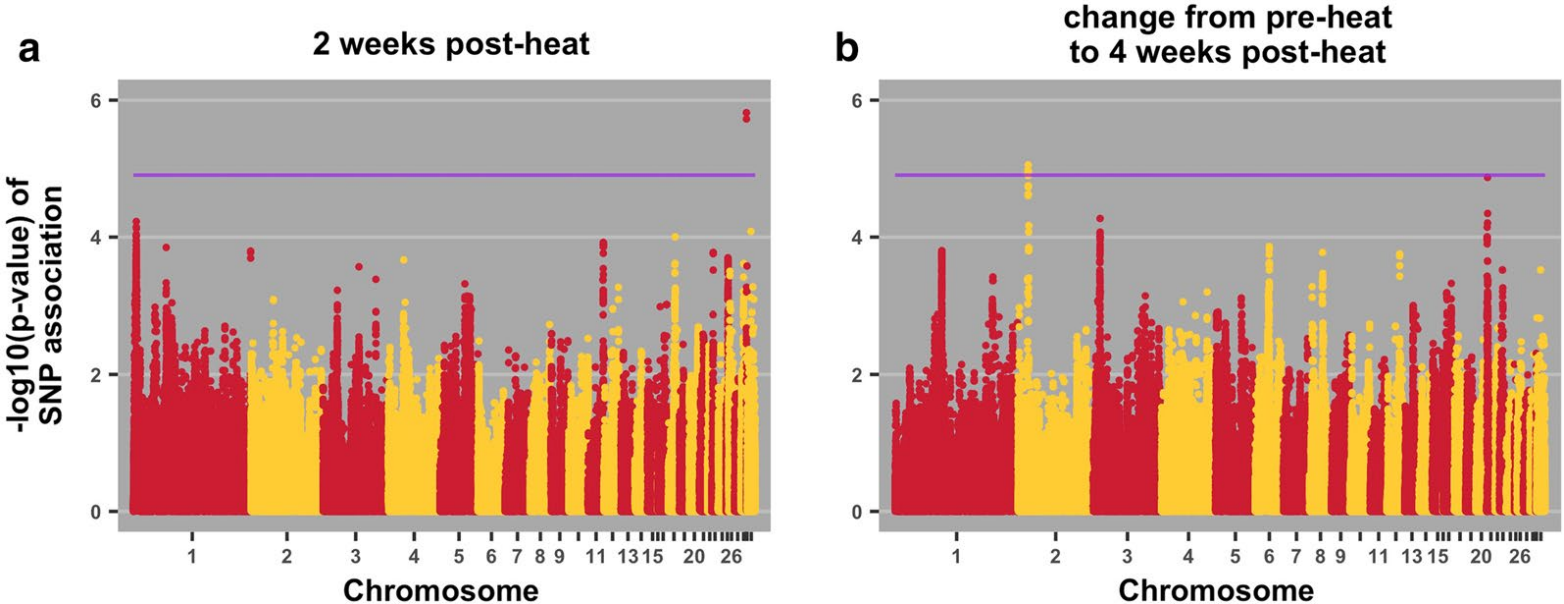
Manhattan plots for egg mass 2 weeks post-heat initiation (**a**) and change from pre-heat to 4 weeks post heat (**b**). The purple line indicates the 20% genome-wide threshold

Quantitative trait loci (QTL) were identified for 10 of the 17 phenotypes that had a heritability higher than 0 (see Figs. 1, 2, 3 and 4). The QTL that reached the 20% genome-wide threshold, the genes that are located within 1 Mb on either side of each SNP, and previously reported relevant QTL associations are listed in Table 4. Details for each individual SNP reaching the 20% genome-wide threshold are in Additional file 1: Table S1.

**Table 4:** QTL associations with traits, positional candidate genes, and previously reported QTL

Trait	Pos^a^	Positional candidate genes and location^b^	Previous relevant QTL associations
Body weight 2 weeks post-heat	18:9	*ENSGALG00000037717*; intron	None
		*SSTR2*; upstream; 111519	
		*SOX9*; downstream; 82531	
Body weight 3 weeks post-heat	18:4	*TNRC6C*; intron	RJF × WL growth rate [32] WL × broiler [41]
		*SEPT9*; downstream; 213717	
		*RAP2B*; downstream; 20951	
Body weight 4 weeks post-heat	18:4	*TNRC6C*; intron	RJF × WL growth rate [32] WL × broiler [41]
		*SEPT9*; downstream; 213717	
		*RAP2B*; downstream; 20951	
	3:57	*TAAR5*; downstream; 744	Broiler × WL, body weight at first egg [31]
		*STX7*; upstream; 7030	
		*VNN1*; downstream; 28285	RJF × WL growth rate [32]
Albumen weight 1 post-week heat	23:5.0	*ENSGALG00000030529*; downstream; 48758	Triglyceride level in broiler × layer cross [42]
		*ADGRB2*; upstream; 2095	
		*gga*-*mir*-*30c*-*1*; downstream; 70983	
		*gga*-*mir*-*1780*; downstream; 68966	
Haugh units pre-heat	5:16.0	*HRAS*; intron	None
		*HRAS*; upstream; 5364	
		*IRF7*; downstream; 184251	
Haugh units 1 week post-heat	2:84	*GALNT1*; intron; 0	Albumen height in non-challenged brown layers [27] Albumen height in non-challenged meat × egg cross at 34 weeks [38] Eggshell thickness [37]
		*gga*-*mir*-*32*; downstream; 366590	
		*INO80C*; downstream; 135338	
		*gga*- *mir*-*187*; downstream; 158132	
		*INO80C*; upstream; 41286	
		*PTPN3*; intron	
		*BAG1*; downstream; 922567	
		*FRRS1L*; upstream; 23398	
		*ENSGALG00000029491*; upstream; 22337	
		*ENSGALG00000033537*; intron	
		*ENSGALG00000029491*; downstream; 31584	
		*ENSGALG00000029935*; downstream; 28455	
		*ENSGALG00000041363*; downstream; 128584	
		*ENSGALG00000033839*; downstream; 102539	
Haugh units 2 weeks post-heat	2:84	*INO80C*; downstream; 218807	Albumen height in non-challenged brown layers [27] Albumen height in non-challenged meat × egg cross at 34 weeks [38]
		*GALNT1*; upstream; 292598	
		*gga*-*mir*-*32*; downstream; 74548	
		*GALNT1*; intron	
		*gga*-*mir*-*32*; upstream; 314649	
		*gga*-*mir*-*187*; downstream; 158132	
		*INO80C*; upstream; 41286	
		*TMEM245*; downstream; 5518	
		*MOCOS*; intron	
		*BAG1*; downstream; 798661	
		*PTPN3*; intron	
		*ENSGALG00000041363*; downstream; 45115	
		*ENSGALG00000033839*; downstream; 102539	
		*ENSGALG00000033537*; intron	
Haugh Units 4 weeks post-heat	5:16	*EFCAB4B*; upstream; 15818	None
		*CD151*; upstream; 45204	
		*PNPLA2*; downstream; 4789	
		*EFCAB4B*; downstream; 1677	
		*H*-*RAS*; intron	
		*H*-*RAS*; upstream; 22102	
		*IRF7*; downstream; 167513	
		*CD151*; intron	
		*RNH1*; upstream; 15030	
		*ENSGALG00000039221*; upstream; 36627	
		*ENSGALG00000041955*; intron	
		*ENSGALG00000038239*; upstream; 113	
		*ENSGALG00000006862*; upstream; 5049	
Egg mass 2 weeks post-heat	33:0.1	*SCN8A*; upstream; 8156	None
		*ENSGALG00000030776*; upstream; 5745	
		*gga*-*mir*-*1668*; downstream; 87357	
Change in egg mass pre-heat to week 4	2:16	*ARHGAP21*; intron; 0	None
		*GPR158*; downstream; 38368	
		*MYO3A*; upstream; 61425	
		*APBB1IP*; upstream; 254361	
		*GPR158*; intron	
		*GPR158*; synon	

^a^Position on chromosome in Mb

^b^Location of SNP relative to neighboring genes (bp)

## Discussion

### Heritability estimates

The moderate heritabilities estimated for body weight (0.31–0.44) before heat exposure and during heat treatment are in agreement with those reported in many other reports for the same trait [19–21] (Table 1). Body weight is generally accepted as a moderately heritable trait. In our study, exposure to heat did not significantly impact heritability estimates for body weight, which is not surprising since the measures were performed on mature hens that are not expected to show significant changes in body weight. In a broiler by Fayoumi cross, Van Goor et al. [22] estimated a heritability of 0.34 for body weight after 1 week of heat challenge.

To our knowledge, this is the first time that estimated heritabilities for albumen weight, Haugh units, and egg mass under heat treatment are reported. For albumen weight, we estimated moderate heritabilities (0.23–0.39) (Table 1), which agree with previous reports, i.e. 0.12–0.59 for albumen weight under normal conditions in various populations [23–26]. For Haugh units, Wolc et al. [27] based on genotyping data reported an estimated heritability of 0.34 in a non-heat-challenged population of brown layers at 26–28 weeks of age (similar to the age (22–28 weeks) of the birds in our study), which is slightly higher than our estimated heritability i.e. 0.26 (Table 1). This difference could be due to the difference in the breed used or the number of observations. Other studies [23, 25, 26] have reported heritability estimates for Haugh units that range from 0.21 to 0.41, which is line with the above results. For egg mass, the estimated heritabilities were moderate (0.24–0.43) and decreased during heat treatment (Table 3).

### Quantitative trait loci

#### Body weight

Three QTL were identified for body weight at three different time points, two on chromosome 18 and one on chromosome 3 (Table 4 and Fig. 1). One of the QTL on chromosome 18 was identified for body weight 2 weeks post-heat. The *SSTR2* gene located near this QTL controls growth hormone secretion [28]. Another QTL on chromosome 18 was detected for body weight at three and at 4 weeks post-heat and the *SEPT9* gene located near this QTL negatively regulates EGFR degradation, which ultimately decreases growth [29]. Down-regulation of growth during a hyperthermic challenge may serve to release resources for more essential, life-sustaining functions or for the reproductive traits for which layer lines are intensively selected. A third positional candidate gene on chromosome 18 near a QTL for body weight, *RAP2B*, protects cells from DNA damage in a p53-dependent manner [30]. Preventing and recovering from DNA damage is a crucial function under hyperthermic conditions. The QTL for body weight, on chromosome 3, has also been associated with body weight in two other independent populations [31, 32]. The *VNN1* gene located near this QTL has a role in lipid metabolism [33].

#### Albumen weight

We identified one QTL on chromosome 23 for albumen weight at one time point, i.e. 1 week after heat initiation (Table 4 and Fig. 2) and one gene, *ADGRB2,* and one microRNA, *gga*-*mir*-*30c*-*1* were located near this QTL. Because neither of these elements has been previously implicated in heat response or egg formation, these are novel associations. Interestingly, *gga*-*mir*-*30c*-*1* was isolated from both the albumen and yolk [34]. The egg is the reproductive unit in chicken and micro RNAs are known to play an important role in gene regulation, thus their existence within the egg suggests that *gga*-*mir*-*30c*-*1* may have a role in embryonic development.

#### Haugh units

Two QTL were identified for Haugh units at four time points (Table 4 and Fig. 3). The QTL on chromosome 5 was found for Haugh units before exposure to heat and at 4 weeks post-heat, which suggests a shared genomic control for these two phenotypes and that genetic selection for Haugh units under normal conditions will also impact Haugh units under a long-term heat treatment. We identified several genes in the vicinity of this QTL and two of these could have a role in Haugh units, i.e. *PNPLA2*, which is upregulated in response to heat [35] and plays a role in hepatic yolk lipoprotein synthesis [36], and *EFCAB4A*, which is involved in calcium ion binding, a crucial function for eggshell formation. The phenotype Haugh units has been reported to be genetically correlated with eggshell characteristics (genetic correlations ranging from 0.13 to 0.36) [23].

The QTL on chromosome 2 was detected for Haugh units at 1 week and at 2 weeks post-heat, which as above suggests a shared genomic control for these two phenotypes. Previously, the region of this QTL has been shown to be associated with eggshell thickness [37] and with albumen height in independent populations of non-heat-challenged hens [27, 38]. Some of the genes located in this region are involved in heat response, i.e. *INO80C* has a role in DNA repair, which is essential in response to a thermal challenge, and *BAG1* and *MOCOS* are known to be downregulated under heat stress [39].

#### Egg mass

Two QTL were identified for egg mass phenotypes (Table 4 and Fig. 4): one on chromosome 33 for average egg mass during the first 2 weeks of heat treatment and one on chromosome 2 for change in average egg mass from prior to heat exposure to the measure at 4-week heat exposure. We found one gene within the region on chromosome 2, *ARHGAP21*, which has been reported to be involved in egg number in geese [40].

## Conclusions

In this study, we quantified phenotypic changes in response to acute and chronic heat exposure in commercial egg laying hens, and found that all the phenotypes were significantly impacted by exposure to high temperature at one or more time points. Seventeen phenotypes had an estimated heritability different from 0, which indicates that they are under genetic control and that there is potential for improving these traits by selective breeding. QTL were identified for 10 of these 17 phenotypes. Some of these phenotypes shared the same QTL across time points, which indicates shared genomic control. Our findings contribute to the knowledge on the genomic control of response to heat stress in laying hens.

## Additional file

**Additional file 1: Table S1.** Trait, position, and p-value information for SNPs reaching the 20% genome-wide threshold.

## Data Availability

Restrictions apply to the availability of these data, which were used under license from Hy-Line International for the current study, and are not publicly available. Data are however available from the authors upon reasonable request and with permission of Hy-Line International.
