# Supplementary material for: Genetic analysis of production, physiological, and egg quality traits in heat-challenged commercial white egg-laying hens using 600k SNP array data
Source: Genet Sel Evol. 2019 Jun 25;51:31. doi: 10.1186/s12711-019-0474-6 (PMC6593552; doi:10.1186/s12711-019-0474-6)
Supplement: Supplementary file 1 — Additional file 1: Table S1. Trait, position, and p-value information for SNPs reaching the 20% genome-wide threshold. [file 12711_2019_474_MOESM1_ESM.pdf]

Table S1: Trait, position, and p-value information for SNPs reaching the 20% genomewide threshold

Trait	SNP	Position[1]	P-value
Albumen weight 1 weeks heat	AX-76293016	23:5018426	5.52e-06
Haugh units pre heat	AX-76783301	5:16033595	1.24e-05
Haugh units 1 week heat	AX-76166420	2:84131913	3.61E-06
	AX-76166253	un-localized	4.46E-06
	AX-76166444	2:84148089	6.56E-06
	AX-76166769	2:84340486	7.06E-06
	AX-76166961	2:84446006	7.06E-06
	AX-76166265	2:84047829	7.16E-06
	AX-76166318	2:84078395	7.16E-06
	AX-76166455	2:84154322	7.16E-06
	AX-76166456	2:84155374	7.16E-06
	AX-76166478	2:84172587	7.16E-06
	AX-76166486	2:84176689	7.16E-06
	AX-80982716	2:84177913	7.16E-06
	AX-76166493	2:84179014	7.16E-06
	AX-80889176	2:84305979	7.16E-06
	AX-76166756	2:84332508	7.16E-06
	AX-76166767	2:84337632	7.16E-06
	AX-76166914	2:84423955	7.16E-06
	AX-76166950	2:84439068	7.16E-06
	AX-76166970	2:84455353	7.16E-06
	AX-76167005	2:84475576	7.16E-06
	AX-76166982	2:84459017	7.34E-06
	AX-76166962	2:84448699	8.47E-06
	AX-76168012	2:84983895	8.71E-06
	AX-76166466	2:84165069	8.77E-06
	AX-76167039	2:84493970	1.01E-05
	AX-80769204	2:84541027	1.06E-05
	AX-76166415	2:84129666	1.08E-05
	AX-76167231	2:84597667	1.08E-05
	AX-76168148	2:85056902	1.11E-05
	AX-76167339	2:84652180	1.21E-05
	AX-76166661	2:84275064	1.23E-05
Haugh units 2 weeks heat	AX-76166914	2:84423955	1.57E-06
	AX-76166253	un-localized	1.96E-06
	AX-76166420	2:84131913	2.23E-06
	AX-76166756	2:84332508	2.60E-06
	AX-76166318	2:84078395	2.84E-06
	AX-76166444	2:84148089	2.86E-06
	AX-76166961	2:84446006	2.87E-06
	AX-76166769	2:84340486	2.87E-06
	AX-76166265	2:84047829	2.96E-06
	AX-76166455	2:84154322	2.96E-06
	AX-76166456	2:84155374	2.96E-06
	AX-76166478	2:84172587	2.96E-06
	AX-76166486	2:84176689	2.96E-06
	AX-80982716	2:84177913	2.96E-06
	AX-76166493	2:84179014	2.96E-06
	AX-80889176	2:84305979	2.96E-06
	AX-76166767	2:84337632	2.96E-06
	AX-76166950	2:84439068	2.96E-06
	AX-76166962	2:84448699	2.96E-06
	AX-76166970	2:84455353	2.96E-06
	AX-76167005	2:84475576	2.96E-06

	AX-76166466	2:84165069	3.01E-06
	AX-76166982	2:84459017	3.29E-06
	AX-76168242	2:85107801	4.04E-06
	AX-76168148	2:85056902	4.38E-06
	AX-76168104	2:85031666	5.70E-06
	AX-76168218	2:85093024	7.49E-06
	AX-76168091	2:85024396	8.19E-06
	AX-76167039	2:84493970	8.28E-06
	AX-80934531	2:84992922	8.44E-06
	AX-76168012	2:84983895	8.89E-06
	AX-76166776	2:84343131	8.94E-06
Haugh Units 4 weeks heat	AX-76782683	5:15753420	1.71E-06
	AX-76782685	5:15754520	1.71E-06
	AX-76782618	5:15725858	4.55E-06
	AX-76783267	5:16016857	4.73E-06
	AX-76782558	5:15699551	5.60E-06
	AX-76782576	5:15707824	5.60E-06
	AX-76783465	5:16148429	7.63E-06
	AX-76782582	5:15710821	8.18E-06
	AX-77282055	5:16119286	8.49E-06
	AX-76783403	5:16123222	8.49E-06
	AX-76783471	5:16151589	1.14E-05
	AX-77282057	5:16147197	1.19E-05
	AX-76783430	5:16133443	1.23E-05
	AX-76783141	5:15959219	1.23E-05
Body weight 2 weeks heat	AX-75913908	18:8951897	5.15e-06
	AX-75913927	18:8956053	1.09e-05
Body weight 3 weeks heat	AX-75896940	18:4086805	4.93e-06
Body weight 4 weeks heat	AX-75896940	18:4086805	4.82e-07
	AX-80956355	18:4078862	6.64e-06
	AX-76508489	3:57039982	1.12e-05
Egg mass 2 weeks heat	AX-76764925	33:129913	1.52e-06
	AX-76764951	33:135041	1.89e-06
Change in Egg mass pre heat to week 4	AX-76046616	2:16822397	8.81E-06
	AX-76045969	2:16459295	1.06E-05
	AX-76045989	2:16472662	1.07E-05
	AX-76046175	2:16585934	1.08E-05
	AX-76045950	2:16449632	1.13E-05
	AX-76046027	2:16500045	1.13E-05
	AX-76046171	2:16583520	1.13E-05
	AX-76046214	2:16605780	1.13E-05
	AX-76045968	2:16458999	1.13E-05
	AX-76045978	2:16467046	1.13E-05
	AX-76045990	2:16473553	1.13E-05
	AX-76046117	2:16552057	1.13E-05
	AX-76046219	2:16607679	1.13E-05
	AX-76046025	2:16497711	1.13E-05
	AX-76046010	2:16488326	1.18E-05

[1]Chromosome:base pair

[2]Location of SNP relative to neighboring genes (base pairs)
